# Entropy Density Acceleration and Minimum Dissipation Principle: Correlation with Heat and Matter Transfer in Glucose Catabolism

**DOI:** 10.3390/e20120929

**Published:** 2018-12-05

**Authors:** Roberto Zivieri, Nicola Pacini

**Affiliations:** 1 Department of Mathematical and Computer Sciences, Physical Sciences and Earth Sciences, University of Messina, Messina 98166, Italy; 2 Laboratory of Biochemistry F. Pacini, Reggio Calabria 89100, Italy; 3 Department of General Surgery and Senology, University Hospital Company, Catania 95124, Italy

**Keywords:** entropy generation, entropy acceleration, minimum energy dissipation principle, glucose catabolism, irreversible reactions, heat transfer, matter transfer, cancer biology, stem cell biology

## Abstract

The heat and matter transfer during glucose catabolism in living systems and their relation with entropy production are a challenging subject of the classical thermodynamics applied to biology. In this respect, an analogy between mechanics and thermodynamics has been performed via the definition of the entropy density acceleration expressed by the time derivative of the rate of entropy density and related to heat and matter transfer in minimum living systems. Cells are regarded as open thermodynamic systems that exchange heat and matter resulting from irreversible processes with the intercellular environment. Prigogine’s minimum energy dissipation principle is reformulated using the notion of entropy density acceleration applied to glucose catabolism. It is shown that, for out-of-equilibrium states, the calculated entropy density acceleration for a single cell is finite and negative and approaches as a function of time a zero value at global thermodynamic equilibrium for heat and matter transfer independently of the cell type and the metabolic pathway. These results could be important for a deeper understanding of entropy generation and its correlation with heat transfer in cell biology with special regard to glucose catabolism representing the prototype of irreversible reactions and a crucial metabolic pathway in stem cells and cancer stem cells.

## 1. Introduction

In Nature, irreversible processes play a crucial role in maintenance of life for their special chemical and physical features [1,2,3,4]. Regarding this, it is well-known that the products of irreversible reactions are characterized by a chemical stability that gives them a temporal stabilization and a precise time arrow. Hence, it is very interesting to understand the thermodynamics of irreversible reactions occurring in minimum living systems investigating the relation between irreversibility and information. The physical quantity able to describe in a unitary way the irreversibility and information in minimum living systems is entropy [5]. In this respect, very recently, it has been proposed a new variable, the entropy of entropy, enabling to measure the complexity for biological systems through the combination of the multiscale entropy analysis and an alternate measure of information, called the superinformation [6]. Within Prigogine’s framework of non-equilibrium thermodynamics, the local entropy production as a function of time is a key quantity strictly related to the energy dissipation of a system that continuously decreases during development, growing and aging [7,8]. 

Among irreversible reactions in minimum living systems, the most representative are the ones occurring in glucose catabolism characterized by both lactic acid fermentation and respiration metabolic pathways. Since the observation of a prevalent lactic acid fermentation in cancer cells, the so-called Warburg effect or aerobic glycolysis, many efforts were done to understand the origin of this behavior in cancer cells and cancer stem cells [9,10,11]. On the other hand, in the last years also the study of the metabolic network and of the mitochondria network have received a special attention [12].

In recent years, the strict connection between thermodynamic irreversibility and information resulting from the strong influence of metabolic patterns on genetic and epigenetic patterns in biological systems, the study of the generation of entropy in normal, cancer and stem cells has also been highlighted and demonstrated [11,12,13,14,15]. Attention should focus not only on cell biology, but also on the thermodynamic description of cells and of the living systems because this different perspective could offer a unitary view of metabolomics, genomics and epigenomics. In other words, the measurement of entropy flow and the study of the thermodynamic behavior of living systems could allow the measurement of a finite quantity represented by entropy, which correlates the many different aspects of life networks. Indeed, all subsystems of the complex cellular building are joined together by a unified thermodynamic time arrow and must follow the same behavior, that is, the global thermodynamic behavior coincides with the one of all the subsystems of the considered object [16,17].

Entropy generation due to heat and matter transfer in cells has been investigated in terms of Prigogine’s rate of entropy production [18]. For example, entropy production may select non-equilibrium states in multi-stable systems [19], while reaction-diffusion thermodynamics and entropy production may constrain organism performance at higher temperatures yielding optimal temperatures at which biochemical reactions occur [20]. Generally, entropy generation occurs in non-equilibrium systems that can be described, for instance, by means of stochastic Langevin differential equation and Fokker-Planck equation [21,22,23]. 

An important debate on this topic is about the possible compatibility of the maximum and the minimum entropy production principle or if one of the two principles could be selected in relation to the boundary conditions [24]. The proof and validity of the maximum entropy production principle was debated so far [25,26,27,28,29,30,31,32,33,34] and, for a part of the community, is still an open question with several attempts to disprove it. However, very recently a convincing argument in its favor in terms of stochastic thermodynamics has solved the apparent contradictions of the maximum entropy production principle and shown that is not incompatible with the principle of minimum entropy production [19]. The latter is fulfilled when thermodynamic systems that are close to equilibrium flow towards the global thermodynamic equilibrium when the rate of entropy density production reaches its minimum value (zero value). This scheme can be applied to any thermodynamic system including irreversible processes in minimum living systems like cells behaving as open systems and reversibly exchanging both energy, in the form of heat, and matter with the intercellular environment. 

Recently, some methods like entropy generation minimization to study heat transfer in thermodynamic systems including living systems have been developed [35,36,37]. In addition, the exergy of non-equilibrium thermodynamic systems, the application of the thermodynamic and biochemical principles to the analysis of the thermo-chemical output generated by the cell in the environment [38,39] and the stochastic analogue of entropy production for a dilute solution of Brownian particles in a fluid of light particles have been studied [40]. Those investigations are corroborated by the emerging field of systems science for an understanding of biological processes as whole systems instead of as isolated component parts including entropy rate of stochastic processes in systems governed by a nonlinear dynamics (Kolmogorov-Sinai entropy) [41].

In our recent works on the subject [42,43], we have studied not only the usual local entropy production defined, in our framework, rate of internal entropy density production (RIEDP) but also the rate of external entropy density production (REEDP) that is related to heat and matter flows from the cell to the environment due to irreversible processes occurring inside cells. Both contributions reduce with increasing time and vanish at global thermodynamic equilibrium. REEDP is usually called in the literature entropy flow or entropy flow rate [7,18,19,22] to indicate the entropy generated by flows of heat and matter either from the system to the environment or from the environment to the system. However, in our framework, restricted to the entropy generation of a single cell, the REEDP is referred to the rate of entropy density production in the intercellular environment due to the irreversible processes occurring inside the cell. In this respect, we have not considered the flows of heat and matter from the environment towards the cell that would correspond to a negative rate for the environment (but positive for the cell) and a resulting negative REEDP. 

We have already proved that both RIEDP and REEDP fulfill Prigogine’s minimum energy dissipation principle [42,43] that corresponds to the minimum rate of entropy production principle. Let us introduce a dissipative function Ψ = *T d*S*_i_*/*dt* where S*_i_* is the entropy of the system with *T* the temperature. We get *d*Ψ/*dt* ≤ 0 that tends to be minimum (zero) in a steady-state (and as a special case of steady state at global thermodynamic equilibrium) [7]. In this study, we prove Prigogine’s minimum energy dissipation principle via the calculation of a novel physical quantity, the time derivative of the rate of entropy density production. This quantity is defined as the entropy density acceleration generated by heat and matter transfer inside cells and with the intercellular environment in irreversible reactions such as the ones characterizing glucose catabolism. The introduction of the entropy density acceleration stems from the mechanical concept of acceleration that plays a crucial role to characterize the dynamics of rigid bodies in kinematics and mechanics. Basing on the analogy between the behavior of mechanical systems and thermodynamic systems, we redefine this concept in out-of-equilibrium thermodynamics. We remind that this concept derived from mechanics and applied to thermodynamics is not an end in itself and the model developed does not represent a mere theorization but tries to describe reality, as it is consistent with the experimental data of several works [11,12,13,14,15]. 

In this way, we reformulate Prigogine’s minimum energy dissipation principle in terms of the entropy density acceleration calculated inside and outside a cell (normal or cancer) performing also the derivative of the REEDP (or entropy flow) and giving a more complete quantitative analysis of out-of-equilibrium processes. In particular, we show that the acceleration both inside and outside the cell approaches zero at global thermodynamic equilibrium. These calculations allow us to understand better the entity of the entropy production at the starting instants of time of the irreversible processes when the system is out-of-equilibrium and to strengthen the principle of minimum entropy production at global thermodynamic equilibrium together with its strict relation with the minimum energy dissipation principle. 

The key result of this work is the rigorous demonstration, by means of analytical and numerical calculations, of the reduction of entropy density acceleration with increasing time and its vanishing at global thermodynamic equilibrium. In particular, we have found that, during heat and matter transfer in either normal or cancer cells, the entropy density “decelerates” due to the negative value of the entropy density acceleration passing, as a function of time, from one local equilibrium state (but global non-equilibrium) to the following local equilibrium state. This “deceleration” approaches zero at large times corresponding to the global thermodynamic equilibrium.

These findings allow us to propose a reformulation of Prigogine’s minimum energy dissipation principle in terms of the vanishing of the entropy density acceleration at global equilibrium and to apply it to the most representative catabolic process occurring in cells, the glucose catabolism. The results obtained for glucose catabolism can be generalized to any kind of irreversible reactions occurring in either normal or cancer cells of living systems.

## 2. Methods

In this section, we derive the general expressions of the entropy density acceleration for glucose catabolism resulting from the rates of entropy density calculated in [42,43] and based on thermodynamic and statistical principles. 

### 2.1. Entropy Density Acceleration for Glucose Catabolism

To calculate the entropy density acceleration for glucose catabolism defined as the time derivative of the rate of entropy density, namely *a*(*x*,*t*) = ∂*r*(*x*,*t*)/∂*t*, we recall the general definition of the rate of entropy density production decomposed as *r*(*x*,*t*) = *r_i_* (*x*,*t*) + *r_e_* (*x*,*t*). Here, *r_i_* = ∂*s_i_*/∂*t* ≥ 0 is the RIEDP with, in a compact form, *s_i_* = *S_i_*/V (*S_i_* is the internal entropy and V is the volume of the thermodynamic system) the internal entropy density, and *r_e_* = ∂*s_e_*/∂*t* is the REEDP with, in a compact form, *s_e_* = *S_e_*/V (*S_e_* is the external entropy) the external entropy density. This latter quantity is often called in the literature the entropy flow or entropy flow rate or entropy production rate of the system [18,19,22]. However, for the sake of simplicity, we have adopted the same nomenclature of the internal contribution.

Note that, in our framework, we have computed all quantities related to the entropy production (forces, heat flow, velocity of reactions, external heat release, internal and external mass flow) during glucose catabolism as referred to a single cell regarded as an open thermodynamic system exchanging heat and matter with the intercellular environment via the cell membrane [42,43]. Heat and matter flow between the cell and the intercellular environment do not include the contributions due to other cells and entering into the cell (taken as reference) but only the ones from the reference cell to the intercellular environment. Hence, a reasonable way to perform the calculation of entropy density acceleration is to divide the entropy acceleration by the volume of the cell for both its internal and external contributions. Of course, a generalization of the model to an agglomerate of cells would imply the computation of the external contribution to the rate of entropy density production taking into account also the volumes of the surrounding cells that face towards the intercellular environment. In this respect, a model grain growth considering a population of grains with finite volume that could recall a population of cells has been developed in a space of grain sizes in the presence of both absorbing and reflecting boundary conditions [44]. 

We remind that, in our previous works on the subject [42,43], we have called REEDP the rate of entropy density contributions expressing the entropy flow rate of heat and matter reversibly exchanged from inside the cell to the intercellular environment and due to irreversible processes occurring inside the cell. This results in an increase of the rate of entropy production in the intercellular environment (external to the system represented by the cell) at the expenses of an equal decrease of the rate in the cell. This means that, if we consider the REEDP or entropy flow rate as referred to a loss of the rate of entropy density production of the cell regarded as the thermodynamic system exchanging entropy with the environment, we should take it with the negative sign as is usually done in the literature [19,22]. However, in our framework according to which we consider the whole thermodynamic system decomposed into two subsystems, the cell and the intercellular environment, the entropy density flow rate should be interpreted as a gain for the intercellular environment and taken with the positive sign and added to the RIEDP.

Of course, when one deals with an open thermodynamic system undergoing state changes due to external driving forces also the entropy flow rate from the environment into the system should be taken into account. At a non-equilibrium steady state, the time averaged entropy change is zero due to the balancing between the entropy flow rate (either from the system to the environment or from the environment into the system) and the rate of internal entropy production [19,22]. However, in our framework, as discussed above we do not deal with driving forces external to the system and consequently with the entropy flow rate from the environment to the system. At global thermodynamic equilibrium, all quantities (rates and accelerations) are zero.

More specifically, in our framework, *r_i_*(*x,t*) = *r_i Q_*(*x,t*) + *r_i D_*(*x,t*) + *r_i r_*(*x,t*), with *r_i Q_*(*x,t*) the contribution due to heat flow and transfer inside the cell, *r_i D_*(*x,t*) the one associated to molecules diffusion and internal transport and *r_i r_*(*x,t*) the one due to irreversible chemical reactions occurring inside the cell. In the special case studied, glucose catabolism occurs in two compartments, cytoplasm and mitochondria characterized by different internal structure morphology. In particular, some reactions occur in the cytoplasm (compartment 1), giving rise to internal entropy production *d*S_1_ and other reactions in the mitochondria (compartment 2), giving rise to internal entropy production *d*S_2_. According to the local formulation of the second principle of thermodynamics, due to the extensive nature of entropy, the total entropy production is *d*S_tot_ = *d*S_1_ + *d*S_2_, that is *d*S_tot_ is the sum of the contributions of its subparts [18]. We have applied this scheme to each internal heat and matter contribution. It was then reasonable to divide each contribution by the average volume of the cell that is different for normal and cancer cells calculating first the rate of internal entropy density production ∂*s_i_*/∂*t* for heat and mass contributions and then the internal entropy density accelerations. Hence, even though it does not appear explicitly, the compartmentalization of the cell has been taken into account in the calculations.

From the rates defined above, we get *a_i_*(*x*,*t*) = ∂*r_i_*(*x*,*t*)/∂*t* for the internal entropy density acceleration (IEDA) with *a_i_*(*x,t*) = *a_i Q_*(*x,t*) + *a_i D_*(*x,t*) + *a_i r_*(*x,t*). Instead, *r_e_*(*x,t*) = *r_e Q_*(*x,t*) + *r_e_*_exch_ (*x,t*) with *r_e Q_*(*x,t*) (*r_e_*_exch_ (*x,t*)) is the contribution due to the heat transfer (matter exchange) from the cell to the intercellular environment. As a result, the external entropy density acceleration (EEDA), *a_e_*(*x*,*t*) = ∂*r_e_*(*x*,*t*)/∂*t* includes two contributions, *a_e_*(*x*,*t*) = *a_e Q_*(*x,t*) + *a_e_*_exch_(*x,t*) where *a_e Q_*(*x,t*) = ∂*r_e Q_*(*x*,*t*)/∂*t* (*a_e_*_exch_(*x,t*) = ∂*r_e_*_exch_(*x*,*t*)/∂*dt*) is the acceleration contribution related to heat (matter) transfer between the cell and the intercellular environment. Therefore, owing to the previous definitions *a*(*x*,*t*) = *a_i_*(*x,t*) + *a_e_*(*x*,*t*) is the total acceleration. We note, according to this framework, that the entropy density acceleration has a space and time dependence and this latter dependence is still one-dimensional as for *r*(*x*,*t*) *= r_i_* (*x*,*t*) + *r_e_*(*x*,*t*).

### 2.2. Internal Entropy Density Acceleration for Glucose Catabolism

In this subsection, we calculate the contributions to the IEDA relevant to glucose catabolism with special regard to lactic acid fermentation and respiration processes. In the calculations, we have taken into account the same assumptions made in our previous studies about the shape of the cells (cubic), the features of the heat and mass flows (unidirectional), the kind of tissue and so on [42,43] based on direct observations for any type of cell (normal or cancer). The IEDA *a_iQ_*(*x,t*) due to heat transfer inside the cell is:

(1) $$a_{i\;Q}\left(x,t\right)=\frac{\partial }{\partial t}\left[\nabla \left(\frac{1}{T\left(x,t\right)}\right)\;\cdot J_{u}\left(x,t\right)\right].$$

Here, $J{\;}_{u}=J_{Q}+\sum_{k=1}^{N}u_{k}J_{Dk}$ with *N* the number of chemical species, $J_{Q}$ is the irreversible heat flow, $J_{Dk}$ is the diffusion flow of the *k*th chemical species, *u_k_* is the partial molar energy and ***F****_u_*(*x*,*t*) = **∇**(1/*T*(*x*,*t*)) is the heat thermodynamic force driving $J_{u}$ with *T*(*x*,*t*) the temperature distribution. Instead, the IEDA due to mass diffusion and matter exchange inside the cell assumes the form:

(2) $$a_{i\;D}\left(x,t\right)=-\frac{\partial }{\partial t}\left[\sum_{k=1}^{N}\nabla \left(\frac{\mu_{k}\left(x,t\right)}{T\left(x,t\right)}\right)\;\cdot \;J_{Dk}\left(x,t\right)\right],$$

where the diffusion flow $J_{Dk}$ is driven by the matter thermodynamic force **F***_k_*(*x*,*t*) = **∇**(*μ_k_*(*x*,*t*)/*T*(*x*,*t*)) with *μ_k_* the chemical potential of the *k*th chemical species with *k* = 1, 2, …, *N*. The entropy density acceleration generated by the chemical irreversible reactions reads:

(3) $$a_{i\;r}\left(x,t\right)=\frac{\partial }{\partial t}\left[\frac{1}{T\left(x,t\right)}\sum_{j=1}^{M}A_{j}\left(x,t\right)V_{j}\right].$$

Here, $A_{j}\left(x,t\right)=-\sum_{k=1}^{N}\nu_{jk}\mu_{k}\left(x,t\right)$ is the affinity of the *j*th chemical reaction (*M* is the number of chemical reactions) with *ν_jk_* the stoichiometric coefficients, and *v_j_* = 1/V_cell_ *dξ_j_*/*dt* is the velocity of the *j*th reaction with *dξ_j_* the variation of the *j*th degree of advancement and V_cell_ = *L*^3^ is the volume of the cubic cell (*L* is the side of the average cube). We now calculate the IEDA *a_i Q_*(*x,t*) = *∂r_i Q_* (*x*,*t*)/*∂t* associated to the heat flow during glucose catabolism recalling the expression of *r_i Q_*(*x,t*) [43]:

(4) $$r_{i\;Q}\left(x,t\right)=p\;K\;\frac{\pi^{2}}{L^{2}}\frac{{\left[\sum_{n=1}^{+\infty }\left(\cos\left[\left(2n-1\right)\frac{\pi }{L}x\right]e^{-\kappa {\left(2n-1\right)}^{2}\frac{\pi^{2}}{L^{2}}t}\right)\right]}^{\;2}e^{-\;\frac{t}{\tau }}}{{\left[\sum_{n=1}^{+\infty }\left(\frac{1}{2n-1}\sin\left[\left(2n-1\right)\frac{\pi }{L}x\right]e^{-\kappa {\left(2n-1\right)}^{2}\frac{\pi^{2}}{L^{2}}t}\right)\right]}^{\;2}}.$$

Here, *K* is the thermal conductivity, *κ* is the thermal diffusivity in water and *τ* is a characteristic decay time. The coefficient *p* = 0.85 (0.90) expresses the frequency of occurrence of glucose catabolism in a normal (cancer) cell. According to the model, we assume that this contribution is the same for lactic acid fermentation and respiration processes. The IEDA associated to heat transfer inside the cell is:

(5) $$\begin{array}{l} a_{i\;Q}\left(x,t\right)=\\ -\frac{p\;K\;\frac{\pi^{2}}{L^{2}}e^{-\;\frac{t}{\tau }}\sum_{n=1}^{+\infty }\left(\cos\left[\left(2n-1\right)\frac{\pi }{L}x\right]e^{-\kappa {\left(2n-1\right)}^{2}\frac{\pi^{2}}{L^{2}}t}\right)}{{\left[\sum_{n=1}^{+\infty }\left(\frac{1}{2n-1}\sin\left[\left(2n-1\right)\frac{\pi }{L}x\right]e^{-\kappa {\left(2n-1\right)}^{2}\frac{\pi^{2}}{L^{2}}t}\right)\right]}^{\;2}}\times \\ \left[\frac{1}{\tau }\left[\sum_{n=1}^{+\infty }\left(\cos\left[\left(2n-1\right)\frac{\pi }{L}x\right]e^{-\kappa {\left(2n-1\right)}^{2}\frac{\pi^{2}}{L^{2}}t}\right)\right]+2\kappa \frac{\pi^{2}}{L^{2}}\left[\begin{array}{l} -\frac{\sum_{n=1}^{+\infty }\left(\left(2n-1\right)\sin\left[\left(2n-1\right)\frac{\pi }{L}x\right]e^{-\kappa {\left(2n-1\right)}^{2}\frac{\pi^{2}}{L^{2}}t}\right)\left[\sum_{n=1}^{+\infty }\left(\cos\left[\left(2n-1\right)\frac{\pi }{L}x\right]e^{-\kappa {\left(2n-1\right)}^{2}\frac{\pi^{2}}{L^{2}}t}\right)\right]}{\left[\sum_{n=1}^{+\infty }\left(\frac{1}{2n-1}\sin\left[\left(2n-1\right)\frac{\pi }{L}x\right]e^{-\kappa {\left(2n-1\right)}^{2}\frac{\pi^{2}}{L^{2}}t}\right)\right]}+\\ +\sum_{n=1}^{+\infty }\left({\left(2n-1\right)}^{2}\cos\left[\left(2n-1\right)\frac{\pi }{L}x\right]e^{-\kappa {\left(2n-1\right)}^{2}\frac{\pi^{2}}{L^{2}}t}\right) \end{array}\right]\right] \end{array}$$

*a_i Q_*(*x*,*t*) includes two terms depending on the trigonometric series: the first term is inversely proportional to the characteristic decay time, while the second one is proportional to the thermal conductivity. 

We now determine the IEDA *a_i D_*(*x,t*) = ∂*r_i D_*(*x*,*t*)/∂*t* either for respiration or fermentation process recalling the corresponding rate of entropy density [43]:

(6) ri D α(x,t)=π32161T01Vcell (x−L/2)e−tτt32(∑k=1Nα(ukNm kDk e−(x−L/2)24Dkt))((∑n=1+∞(cos[(2n−1)πLx]e−κ(2n−1)2π2L2t)e−|x−L/2|/L)(∑n=1+∞(12n−1sin[(2n−1)πLx]e−κ(2n−1)2π2L2t))2) .

Here, *α* = ferm, resp where “ferm” stands for fermentation and “resp” for respiration, *T*_0_ is the maximum cell temperature, *N_α_* is the number of chemical species in either the respiration or fermentation process, *D_k_* is the diffusion constant of the *k*th chemical species, *μ_k_* = *u_k_*_∙_*_e_*^−(|*x* − *L*/2|/*L* + *t*/^^τ)^ is the *k*th species chemical potential, and *N_m k_* is the number of moles of the *k*th chemical species. The IEDA associated to matter diffusion inside the cell either for respiration or fermentation process reads:

(7) ai D α(x,t)=−π32161T01Vcell(321t52+1τ1t32)(x−L/2)e−tτ[(∑k=1Nα(ukNm kDk e−(x−L/2)24Dkt))((∑n=1+∞(cos[(2n−1)πLx]e−κ(2n−1)2π2L2t)e−|x−L/2|/L)(∑n=1+∞(12n−1sin[(2n−1)πLx]e−κ(2n−1)2π2L2t))2)]++π32161T01Vcell  (x−L/2)3e−tτ t32 (∑k=1Nα(ukNm kDk 14Dkt2e− (x−L/2)24Dkt))((∑n=1+∞(cos[(2n−1)πLx]e−κ(2n−1)2π2L2t)e−|x−L/2|/L)(∑n=1+∞(12n−1sin[(2n−1)πLx]e−κ(2n−1)2π2L2t))2)++π32161T01Vcell (x−L/2)e−tτ t32[∑n=1+∞(12n−1sin[(2n−1)πLx]e−κ(2n−1)2π2L2t)] 2(∑k=1Nα(ukNm kDk e−(x−L/2)24Dkt))×2κπ2L2e−|x−L/2|/L[∑n=1+∞((2n−1)sin[(2n−1)πLx]e−κ(2n−1)2π2L2t)[∑n=1+∞(cos[(2n−1)πLx]e−κ(2n−1)2π2L2t)][∑n=1+∞(12n−1sin[(2n−1)πLx]e−κ(2n−1)2π2L2t)]−∑n=1+∞((2n−1)2cos[(2n−1)πLx]e−κ(2n−1)2π2L2t)]. 

*a_i Dα_* (*x,t*) consists of three contributions each of them weighted by the trigonometric series.

Taking into account the weights *w*_ferm_ and *w*_resp_ expressing the frequency of occurrence of respiration and fermentation process in a normal and in a cancer cell (see Section 3, Results), we write the total acceleration contribution *a_i D_* due to the two metabolic pathways as:

*a_i D_*(*x,t*) = *w*_resp_*a_i D_*_resp_ (*x,t*) + *w*_ferm_*a_i D_*_ferm_ (*x,t*) (8)

Finally, we compute the IEDA *a_i r_*(*x,t*) = ∂*r_i r_* (*x*,*t*)/∂*t* caused by irreversible reactions occurring inside the cell during glucose catabolism via the corresponding rate expressed in the form [43]:

(9) ri r α(x,t)=−π41T0 1Vcelle−t/τ kkin α (∑k=1Nανk uke−|x−L2|/L Nm Glucose)∑n=1+∞(sin[(2n−1)πLx]2n−1e−κ(2n−1)2π2L2t),

with *k*_kin_ *_α_* the kinetic constant, *ν_k_* stoichiometric coefficients and *N_m_*_Glucose_ the number of glucose moles (note the typo error *L*/2 – *x* in the argument of the sine in Equations (4), (10) and (11) of [43]). We get *a_i r_*(*x,t*) either for respiration or for lactic acid fermentation:

(10) ai r α(x,t)=−π41T0 1Vcelle−t/τ kkin α∑k=1Nανk uke−|x−L2|/L Nm Glucose× [−1τ(∑n=1+∞(sin[(2n−1)πLx]2n−1e−κ(2n−1)2π2L2t))−1+κπ2L2∑n=1+∞((2n−1)sin[(2n−1)πLx]e−κ(2n−1)2π2L2t
) (∑n=1+∞(sin[(2n−1)πLx]2n−1e−κ(2n−1)2π2L2t))2].

*a_i r__α_* (*x,t*) consists of three contributions. We express the entropy density acceleration due to irreversible reactions during glucose catabolism in the form:

*a_i r_* = *w*_resp_*a_i r_*_resp_ + *w*_ferm_*a_i r_*_ferm_, (11)

with different weights for respiration and fermentation process (see Section 3, Results).

### 2.3. External Entropy Density Acceleration for Glucose Catabolism

In this subsection, we calculate the external entropy density acceleration (EEDA) starting from the corresponding REEDP calculated in [42,43]. The EEDA *a_e_*(*x*,*t*) = ∂*r_e_*(*x*,*t*)/∂*t* includes two contributions, namely *a_e_*(*x*,*t*) = *a_e Q_*(*x,t*) + *a_e_*_exch_(*x,t*). Here, *a_e Q_*(*x,t*) = ∂*r_e Q_*(*x*,*t*)/∂*t* (*a_e_*_exch_(*x,t*)= ∂*r_e_*_exch_(*x*,*t*)/∂*t*) is the acceleration contribution related to heat (matter) transfer between the cell and the intercellular environment. The general expression for *a_e Q_*(*x,t*) reads:

(12) $$a_{e\;Q}\left(x,t\right)=\frac{\partial }{\partial t}\left(\frac{1}{T_{ic}\left(x,t\right)}\frac{1}{V_{\mathrm{cell}}}\frac{dQ}{dt}\right),$$

where *T*_ic_ is the intercellular temperature and *dQ*/*dt* is the time derivative of the heat. Instead, the general expression for *a_e_*_exch_ takes the form:

(13) $$a_{e\;\mathrm{exch}}\left(x,t\right)=-\frac{\partial }{\partial t}\left(\frac{1}{T_{ic}\left(x,t\right)}\sum_{k=1}^{N_{\mathrm{pr}}}\mu_{k}\left(x,t\right)\;\frac{d_{e}N_{m}{\;}_{k}}{dt}\right),$$

where *N*_pr_ is the number of products of the reaction and *N_m k_* is the number of moles of the *k*th product of reaction and.

To calculate the *a_e Q_* contribution to EEDA, we recall the corresponding REEDP for respiration and fermentation process [43]:

(14) $$r_{e\;Q}^{{}_{}}{\;}_{\alpha }\;\left(x,t\right)=\frac{1}{V_{\mathrm{cell}}}\frac{3}{8}k_{B}N_{A}N_{m\;\mathrm{pr}\;\alpha }\left[\frac{1\;}{\kappa }\frac{{\left(x-L\right)}^{\;2}}{\;t{\;}^{2}}-\frac{2}{t}\right],$$

where, *k*_B_ = 1.3805 × 10^−23^ J/K is the Boltzmann constant, *N_m_*_pr resp_ (*N_m_*_pr ferm_) is the number of moles of the products in respiration (fermentation) process. Note the contribution proportional to 1/*t* on the second member that breaks the time reversal symmetry [42] as occurs for the other rate contributions. In principle, at small distances from the border of the cell (for small *x*) the term proportional to 1/*t* is greater than the term proportional to 1/*t*^2^ resulting in a negative *r_e Q_* for the typical intercellular size. However, in our model, we have set ourselves under the hypothesis of large *x* and small *t* (1000 μs << 1 s) neglecting the term proportional to 1/*t* even close to the cell border yielding:

(15) $$r_{e\;Q\;\alpha }^{{}_{}}\left(x,t\right)≃\frac{1}{V_{\mathrm{cell}}}\frac{3}{8}k_{B}N_{A}N_{m\mathrm{pr}\;\alpha }\frac{1\;}{\kappa }\frac{{\left(x-L\right)}^{\;2}}{\;t{\;}^{2}}.$$

This assumption has allowed us to get a positive *r_e Q_* for any *x* outside the cell and any *t*. This means that the entropy outside the cell increases because of the heat released by the irreversible reactions occurring inside the cell but this increase leads to a decrease of the rate of entropy of the cell because heat is removed from the cell. Hence, the EEDA *a_e Q_*(*x,t*) takes the simple form:

(16) $$a_{e\;Q\;\alpha }^{}\left(x,t\right)≃-\frac{1}{V_{\mathrm{cell}}}\frac{3}{8}k_{B}\frac{N_{A}N_{m\;\mathrm{pr}\;\alpha }}{\kappa }\frac{{\left(x-L\right)}^{\;2}}{\;t{\;}^{3}}.$$

The total entropy density acceleration reads:

(17) $$a_{e\;Q\;}^{}=w_{\mathrm{resp}}a_{e\;Q\;\mathrm{resp}}^{}+w_{\mathrm{ferm}}\;a_{e\;Q\;\mathrm{ferm}}^{},$$

with different weights for a normal and a cancer cell (See Section 3, Results).

Let us now calculate the last entropy acceleration contribution caused by irreversible exchange of matter. To do this we recall the REEDP due to irreversible exchange of matter with the intercellular environment during glucose catabolism [43]:

(18) re exch α(x,t)=−1T0 1Vcell4π κ t x0e(x−L)24κ te− t/τ∑k=1Npr αuk e−|x−L2|/L deNm k αdτβ.

Here, *x*_0_ = 10 μm is a characteristic length having the size of a normal cell, *dτ_β_* (*β* = 1,2 with 1 referred to respiration and 2 to fermentation) is a characteristic time such that 1/*dτ*_1_ (1/*dτ*_1_) is 10^−5^ s^−1^ (10^−4^ s^−1^) of the order of *k*_kin_, *N*_pr resp_ (*N*_pr ferm_) is the number of products of the respiration (fermentation) process. Also this matter contribution has a positive contribution if referred to the intercellular environment but it would be taken as negative if referred to the cell because matter exchange leads to a removal of the products of irreversible reactions from the cell.

The EEDA *a_e_*_exch_ *_α_* (*x,t*) = *∂r_e_*_exch_ (*x*,*t*)/*∂t* resulting from irreversible exchange of matter with the intercellular environment turns out to be:

(19) ae exch α(x,t)=−1T0 1Vcell1 x0e(x−L)24κ te− t/τ(−1τ4π κ t+π κ t−12πκ(x−L)2t32) ∑k=1Npr αuk e−|x−L2|/L deNm k αdτβ.

*a_e_*_exch_ *_α_* (*x,t*) consists of three contributions. We calculate the entropy acceleration related to exchange of matter with the intercellular environment as:

*a_e_*_exch_ = *w*_ferm_*a_e_*_exch ferm_ + *w*_resp_*a_e_*_exch resp_, (20)

with different weights for a normal and a cancer cell (See Section 3, Results).

## 3. Results 

In this section, we compute the IEDA and the EEDA generated by inner and outer heat and matter transfer in a normal and a cancer cell during glucose catabolism by performing the numerical derivatives of the corresponding RIEDP and REEDP whose analytical expressions were given in Section 2. Indeed, the graphical representation of the analytical formulae of the IEDA would lead to some convergence problems due to their expressions involving several trigonometric series depending on the index *n*. 

We first determine the entropy density acceleration taking into account the occurrence of both respiration and fermentation metabolic pathways occurring with different weights in a normal and cancer cell. Then, we single out the entropy density acceleration due to either respiration or lactic acid fermentation in a representative cell having the size of a normal cell. Cell respiration consists of three steps, viz. glycolysis, Krebs cycle and oxidative phosphorylation (OXPHO) involving glucose and oxygen, C_6_H_12_O_6_ + 6O_2_ → 6 CO_2_ + 6 H_2_O and leading to the formation of carbon dioxide (CO_2_) and water (H_2_O). Instead, fermentation process or aerobic glycolysis consists only of glycolytic step, C_6_H_12_O_6_ → 2 C_3_H_5_ O_3^−^_ + 2 H^+^ and leads to the formation of lactic acid ions (C_3_H_5_ O_3^−^_) and hydrogen ions (H^+^).

For consistency, in the numerical calculations of acceleration densities, we have employed the same numerical parameters used in our previous studies [42,43]. In particular, we have taken an average size *L* = 10 μm (*L* = 20 μm) for the normal cell (cancer cell) taking as reference the breast epithelium and assuming, without loss of generality, a cubic shape and an average size of the intercellular space about 0.2–0.3 μm (1.5 μm) between two adjacent normal (cancer) cells. For all acceleration entropy densities, we have chosen *τ* ≈ 10^−4^ s as a typical cell decaying time. We have taken as weights for respiration (fermentation) process *w*_resp_ = 0.8 (*w*_ferm_ = 0.2) in a normal cell and *w*_resp_ = 0.1 (*w*_ferm_ = 0.9) in a cancer cell. We have also used for both normal and cancer cells the following parameters: thermal conductivity *K* = 0.600 J/(m s K), thermal diffusivity in water *κ*_H20_ = 0.143 × 10^−6^ m^2^/s, diffusion constants at standard conditions: *D*_C6H12O6_ = 6.73 × 10^−10^ m^2^s^−1^, *D*_O2_ = 21.00 × 10^−10^ m^2^s^−1^, *D*_CO2_ = 19.20 × 10^−10^ m^2^s^−1^, *D*_H2O_ = 21.00 × 10^−10^ m^2^s^−1^, *D*_C3H5O3−_ = 9.00 × 10^−10^ m^2^s^−1^ and *D*_H+_ = 45.00 × 10^−10^ m^2^s^−1^ in aqueous solution. Finally, we have employed the partial molar energies or chemical potentials at *t* = 0 and *x* = *L*/2 and, at standard conditions: *μ*_C6H12O6_ = −917.44 kJ/mole, *μ*_O2_ = 16.44 kJ/mole, *μ*_CO2_ = −385.99 kJ/mole, *μ*_H2O_ = −237.18 kJ/mole, *μ*_C3H5O3−_ = −516.72 kJ/mole where C_3_H_5_O_3^−^_ is the lactate ion and *μ*_H+_ = 0 kJ/mole in aqueous solution.

In particular, for the calculation of *a_i Q_* we have taken as frequency of occurrence of glucose catabolism the value *p* = 0.85 (0.90) for normal (cancer) cells, while for the calculation of *a_i r_* we have taken as values of the pathway kinetic constants *k*_kin_^resp^ = 10^−5^ s^−1^ and *k*_kin_^ferm^ = 10^−4^ s^−1^, and *N_m_*_Glucose_ = 1 as a reference concentration. 

### 3.1. Entropy Density Accelerations for Normal and Cancer Cells: Numerical Calculations

In this study, we show the numerical calculations of the IEDA and EEDA for normal and cancer breast cells. For every term, we represent the corresponding acceleration entropy density as a function of the spatial coordinate *x* and of the time coordinate *t*. We choose for all accelerations a time interval Δ*t* =1000 μs, a Δ*t* typical of most biological processes [43]. 

Note that in the main panels we plot all the entropy density accelerations in the time interval 100–1000 μs with the exception of *a_e Q_*. Indeed, in the first instants of time (0–100 μs), some terms of the entropy density acceleration are positive as depicted in the insets and, due to the appreciable magnitude, this would mask the leading negative trend of the accelerations in the time interval 100–1000 μs. The positive trend of most of the accelerations during the first instants of time is not surprising and is due to the initial increasing behavior of the corresponding rates. Of course, this behavior is only secondary to the leading and most important negative trend characterizing all the entropy density accelerations.

### 3.2. Internal Entropy Density Acceleration: Numerical Calculations

**Figure 1:**
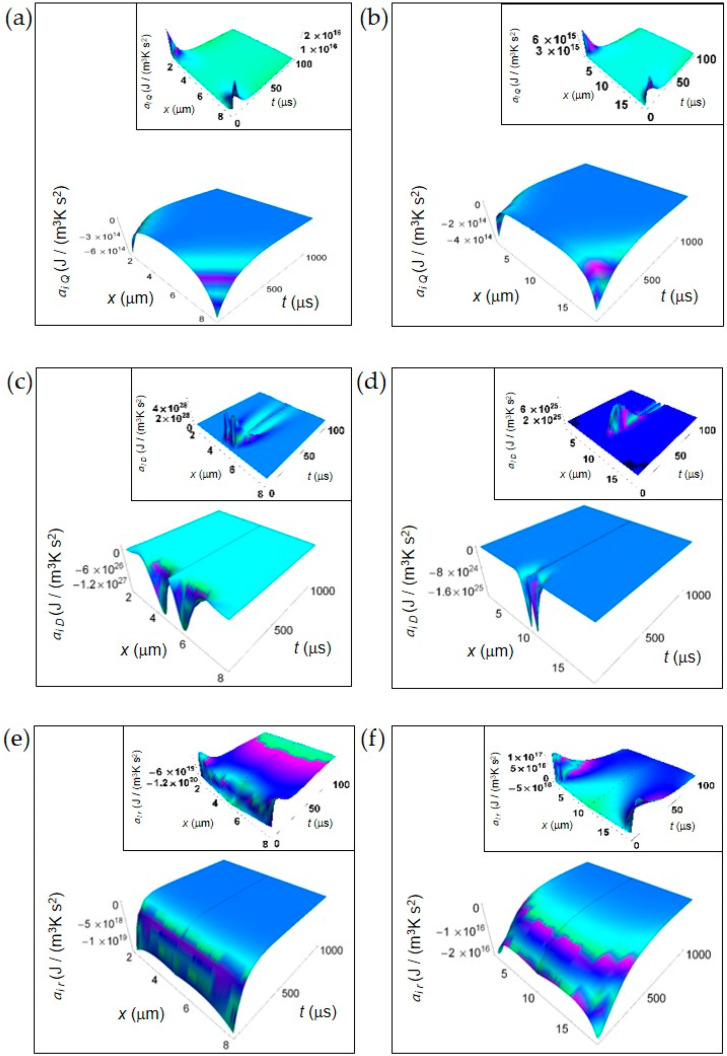
IEDA generated by heat, matter and irreversible reactions during glucose catabolism for a time interval of 1000 μs. (**a**) Calculated *a_i Q_* for a normal cell. Inset: calculated *a_i Q_* for a normal cell in the interval 0–100 μs. (**b**) As in panel (**a**) but for a cancer cell. (**c**) Calculated *a_i D_* for a normal cell. Inset: calculated *a_i D_* for a normal cell in the interval 0–100 μs. (**d**) As in panel (**c**) but for a cancer cell. (**e**) Calculated *a_i r_* for a normal cell. Inset: calculated *a_i r_* for a normal cell in the interval 0–100 μs. (**f**) As in panel (**e**) but for a cancer cell.

In this section, we show the spatial and time dependence of the IEDA obtained from the previous formalism. In Figure 1, we display the IEDA space and time profiles for both normal and cancer cells resulting from the heat and matter transfer inside cells and from the irreversible chemical reactions in the representative time interval 100–1000 μs. The common features are: (1) the negative value of the IEDA corresponding to a “deceleration” and (2) their increase with increasing time. Figure 1a,b shows *a_i Q_* calculated according to Equation (5) for a normal and a cancer cell, respectively. For both kinds of cells, *a_i Q_* dramatically increases with time close to the cell borders, while in the region close to the cell center exhibits a weak increase and an almost flat profile especially in the cancer cell. For *t* > 500 μs the spatial and time profile of *a_i Q_* is rather flat passing from the borders to the cell center and approaches zero with increasing time. 

**Figure 2:**
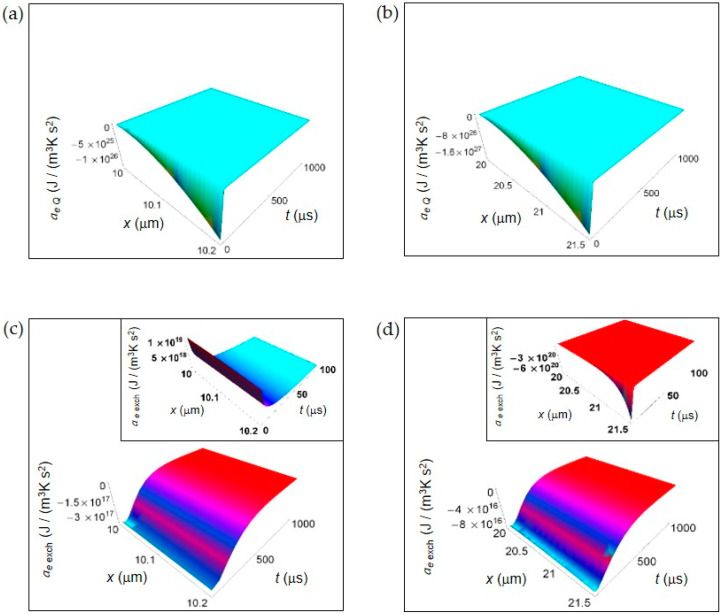
EEDA associated to heat and matter transfer between the cell and the intercellular envinronment. (**a**) Calculated *a*_e *Q*_ for a normal cell. (**b**) As in (**a**), but for a cancer cell. (**c**) Calculated *a_e_*_exch_ for a normal cell. Inset: calculated *a_e_*_exch_ for a normal cell in the interval 0–100 μs. (**d**) As in (**c**), but for a cancer cell.

In contrast, *a_i D_* obtained from Equations (7) and (8) exhibits a strong increase in the central region of the cell for the initial instants of time in both kinds of cells tending to zero for increasing time in the whole cell and exhibiting a flat profile (Figure 2c,d). Note the narrower shape of *a_i D_* in a cancer cell with respect to that in a normal cell, its higher rate of increase at the initial instants of time and a minimum value that is two orders of magnitude less than that of a normal cell. We attribute the general trend to the prevalence of the fermentation process in the cancer cell, while the lesser deep minimum is related to the bigger size of the cancer cell.

Finally, in Figure 1e,f we depict *a_i r_* computed according to Equations (10) and (11). *a_i r_* uniformly increases throughout the whole cell with increasing time but the rate of increase is much higher in a normal cell with respect to a cancer cell. Indeed, for a normal cell *a_i r_* approaches values close to zero for *t* less than 500 μs, while for a cancer cell this occurs for *t* more than 500 μs. This slower tendency towards zero in a cancer cell could be due to the prevalence of lactic acid fermentation. Moreover, the minimum value of *a_i r_* in a cancer cell is about three orders of magnitude less than that of a normal cell and this is in part due to the bigger size of the cancer cell. 

In the insets of Figure 1, we have plotted the IEDA for the initial instants of time (interval 0–100 μs). Interestingly, every contribution is positive with the exception of *a_i r_* for a normal cell that is negative throughout the whole cell and *a_i r_* for a cancer cell that is negative especially in the central part of the cell. In particular, *a_i Q_* for both a normal and a cancer cell and *a_i r_* for a cancer cell exhibit positive values close to the cell borders, while *a_i D_* exhibits remarkable positive values close to the cell centre with some differences as a function of time between a normal and a cancer cell. The positive trend of these contributions reflects the increase of the corresponding rates at the first instants of time. The positive behavior of *a_i r_* in a cancer cell close to the cell borders could be due to the prevalence of the fermentation process with respect to the respiration process.

### 3.3. External Entropy Density Acceleration: Numerical Calculations

In this section, we show the spatial and time dependence of the EEDA obtained from the previous formalism.

Figure 2 shows the EEDA spatial and time profiles generated by heat and matter transfer from the cell to the intercellular environment. In Figure 2a,b we depict the calculated *a_e Q_* for a normal and a cancer cell, respectively calculated according to Equations (16) and (17). For this entropy density acceleration, we have performed the numerical calculations taking the interval of time 0–1000 μs because *a_e Q_*, unlike the other contributions, does not exhibit a positive trend during the interval of time 0–100 μs. 

The more one gets away from the border of the cell, the more the trend of entropy density acceleration becomes sharp exhibiting a strong increase during the initial instants of time that is very similar both in a normal and in a cancer cell. After the initial instants of time, the spatial and time profile of *a_e Q_* becomes flat tending to vanish with increasing *t*. 

In Figure 2c,d is displayed the *a_e_*_exch_ for a normal and a cancer cell calculated according to Equations (19) and (20). The general trend is a uniform increase throughout the intercellular environment during the first instants of time. A sharper increase of *a_e_*_exch_ characterizes the cancer cell because of the prevalence of the fermentation process. However, on average the absolute value of *a_e_*_exch_ for a cancer cell is less than for a normal cell. For *t* larger than 500 μs, in both cases *a_e_*_exch_ approaches zero with increasing time. In the insets of Figure 2c,d, *a_e_*_exch_ plotted in the first instants of time (interval 0–100 μs) shows an opposite behavior taking positive values for a normal cell, and negative values for a cancer cell. This is not surprising and may be attributed to the different size of the cells.

**Figure 3:**
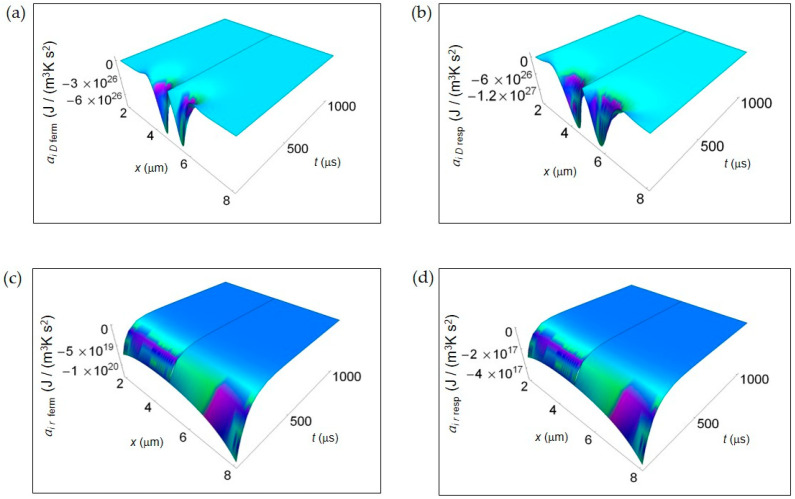
IEDA associated to matter transfer inside the cell for lactic acid fermentation and respiration. A representative cell having the size of a normal cell is depicted. (**a**) Calculated *a_i D_* for fermentation process. (**b**) As in (**a**), but for respiration process. (**c**) Calculated *a_i r_* for fermentation process. (**d**) As in (**c**), but for respiration process.

Figure 3 shows the IEDA spatial and time profiles (time interval 100–1000 μs) due to matter transfer under the hypothesis of either fermentation or respiration metabolic pathways inside a representative cell having the size of a normal cell. In Figure 3a,b, we depict *a_i D_* resulting from lactic acid fermentation and respiration processes, respectively and calculated by means of Equation (7). In the first instants of time, there is a strong rate of increase of *a_i D_* in the central region of the cell for both processes where *a_i D_* is strongly negative. However, there is a broader spatial and time dependence of *a_i D_* for respiration leading to a more extended region of the cell having negative *a_i D_* for small *t*. In addition, also the minimum of *a_i D_*, symmetric on the left and on the right of the center of the cell, is deeper for respiration. For both processes with increasing time *a_i D_* becomes flat and approaches zero. Figure 3c,d displays the corresponding *a_i r_* of the metabolic pathways calculated via Equation (10). Unlike *a_i D_*, there are not relevant differences in the spatial trends of *a_i r_* in the two processes that are uniformly negative throughout the cell even though the minimum for fermentation is much deeper than that for respiration. This trend is in part due to the kinetic constant *k*_kin_^resp^ that is one order of magnitude less than *k*_kin_^ferm^. 

**Figure 4:**
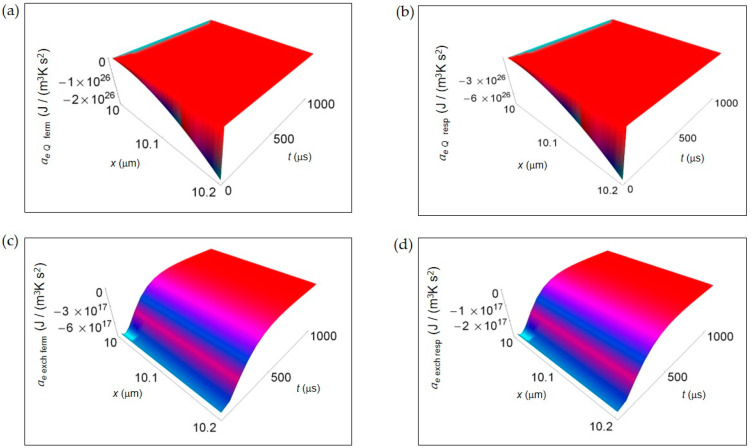
EEDA associated to heat and matter transfer from inside the cell to the intercellular environment for lactic acid fermentation and respiration. A representative cell having the size of a normal cell is depicted. (**a**) Calculated *a_e Q_* for fermentation process. (**b**) As in (**a**), but for respiration process. (**c**) Calculated *a_e_*_exch_ for fermentation process. (**d**) As in (**c**), but for respiration process.

In Figure 4, we represent the EEDA spatial and time profiles (time interval 100–1000 μs) due to heat transfer and mass exchange from inside the cell to the intercellular environment for the two metabolic pathways. Figure 4a,b shows *a_e Q_* for lactic acid fermentation and respiration, respectively calculated using Equation (16). In both cases *a_e Q_* exhibits a deep negative minimum the more the distance from the cell border that is of the same order of magnitude but more pronounced for respiration process. The rate of increase of *a_e Q_* with increasing time is the same approaching zero uniformly in space still at the initial instants of time. In Figure 4c,d, *a_e_*_exch_ computed according to Equation (18) is displayed for lactic acid fermentation and respiration. Like for *a_e Q_* the order of magnitude of the negative minimum is the same but, with increasing the distance from the cell border, the trend of *a_e_*_exch_ remains uniform for both processes. More specifically, the rate of increase of *a_e_*_exch_ is slightly higher for lactic acid fermentation even though, at *t* > 500 μs, *a_e_*_exch_ becomes flat and tends to vanish for both processes. 

In Figure 3 and Figure 4, we have not shown the trend of the IEDA due to matter transfer for the two main metabolic pathways during the initial time interval 0–100 μs. Indeed, the main features of the entropy accelerations in this time interval are very similar to those exhibited for the real glucose catabolism process where there is a mixture of lactic acid fermentation and respiration.

## 4. Discussion

The classical thermodynamic description of glucose catabolism in normal and cancer cells and of fermentation and respiration processes by using the concept of entropy density acceleration allows us to add further important results on irreversible processes in living systems [42,43]. In particular, the analysis extended to the second order in time has strengthened the results obtained in relation to Prigogine’s minimum dissipation principle formulated for living systems in terms of the rate of entropy density. This has been accomplished transferring from mechanics to statistical thermodynamics the concept of “motion” and of acceleration of entropy. We have found that the “motion” characterizing the entropy density in minimum living systems represented by either normal or cancer cells and associated to glucose catabolism and, more specifically, to lactic acid fermentation and respiration processes can be described as a decelerated “motion” because of the negative IEDA and EEDA. 

Note, however, the exceptions represented by the trends of *a_i Q_* and *a_i D_* for both kinds of cells, *a_e_*_exch_ for a normal cell and *a_i r_* for a cancer cell during the first instants of time (interval 0–100 μs), where the entropy density accelerations are positive because of the peculiar time behavior of the terms contributing to these accelerations for small *t*. 

From the inspection of the trend of the out-of-equilibrium IEDA and EEDA, the entropy density acceleration has a remarkable magnitude because of the strong variation as a function of time of the corresponding rates [43]. As a further confirmation of our previous findings where it was found that the entropy gain per unit time was higher during lactic acid fermentation, the total acceleration during lactic acid fermentation has a more pronounced minimum if compared to the corresponding one exhibited by the total acceleration during respiration. We attribute this behavior especially to the *a_i r_* contribution that, for the same cell size taken as reference, is a few orders of magnitude (see Figure 3c) larger for lactic acid fermentation process than for respiration and also to the *a_e_*_exch_ contribution that exhibits a uniform minimum about three times larger for lactic acid fermentation. Indeed, due to the bigger volume of a cancer cell (on average 8 times the one of the normal cell) and to the spatial dependence along *x* that in a cancer cell is twice the one of the normal cell, *a_i r_* and *a_e_*_exch_ shown in Figure 1f and Figure 2d, respectively look only apparently of smaller magnitude in a cancer cell than in a normal cell. This finding reiterates the concept that cancer cells, where lactic acid fermentation prevails, are characterized by a higher entropy gain per unit time (rate of entropy) as found in [43] and, therefore, by a bigger negative entropy acceleration during the initial instants of time. 

Very close to the global thermodynamic equilibrium, Prigogine’s minimum energy dissipation principle is fulfilled and can be reformulated in terms of the minimization of the entropy density acceleration for large times. More specifically, the total entropy density acceleration *a*(*x*,*t*) exhibits a rather out-of-equilibrium deep negative minimum, reduces its magnitude passing through negative values with increasing time and, at the global thermodynamic equilibrium, equals zero independently of the nature of the cell and of the metabolic pathway. Straightforwardly, from the spatial and time profile of the entropy density acceleration, the spatial profile of the energy dissipation function could be obtained, showing that it tends to a minimum value (zero) approaching global thermodynamic equilibrium. 

We believe that the study of the space and time behavior of this thermodynamic quantity could enable to understand more in depth the entropy exchanges in minimum living systems and to give more details on the out-of-equilibrium thermodynamics of lactic acid fermentation and respiration. The notion of entropy density acceleration is easily generalizable to other irreversible reactions occurring in cells and this could give a comprehensive characterization of the thermodynamics of all irreversible processes. 

Finally, the theoretical findings of this work could pave the way to further experiments. We suggest a practical way to measure the entropy production in a biological system in terms of heat and matter flows and, as a result, also the rate of entropy density and the entropy density acceleration theoretically investigated in this work. The heat flow could be determined by using the direct microcalorimetric or modern omics techniques, while a measurement both in vivo and in vitro of the number of moles involved in the lactic acid fermentation or respiration processes via imaging techniques by means of Nuclear Magnetic Resonance or Positive Emission Tomography would be essential for determining the mass flow [42]. This could allow discerning quantitatively the entropy production due to the heat and mass exchanges in normal cells and in stem cells/cancer stem cells confirming, for example, that the entropy density production in these types of cells is higher with respect to the one in normal cells. In this respect, in stem cells, the ratio of entropy production between OXPHO and aerobic glycolysis is different with respect to that of normal cells and cancer cells and the cell volume of stem cells is smaller than that of normal and cancer cells. 

To perform a completely realistic comparison between calculations and measurements, a generalization of the model formulated for a single cell to an agglomerate of cells or a tissue would be necessary. Anyway, the results of the theoretical analysis carried out in this work with special regard to the thermodynamic characterization of fermentation and respiration metabolic pathways are consistent with several works in the literature [13,15].

## 5. Conclusions

In conclusion, the thermodynamics of minimum living systems with special regard to irreversible reactions occurring during glucose catabolism has been theoretically studied via the introduction and the detailed calculation of a quantity directly derived from the well-known rate of entropy density production that we named entropy density acceleration. This latter is defined as the time derivative of the rate of entropy density production and expresses the time behavior at the second order of the entropy generated inside and outside the cell. This was accomplished basing on the idea that a mechanical concept like the one of acceleration may be transferred from mechanics to thermodynamics enabling to understand better the entropy generation caused by heat and matter transfer in turn due to irreversible processes in normal and cancer cells. Owing to this, the well-known Prigogine’s minimum energy dissipation principle at global thermodynamic equilibrium is reformulated in terms of the vanishing of the entropy density acceleration. The advantage of this approach is that it is possible to determine quantitatively the curvature of the rate of entropy density in out-of-equilibrium states not only for glucose catabolism where lactic acid fermentation and respiration processes take place in different percentages in normal and cancer cells but also focusing on the specific metabolic pathway. This has allowed to confirm that lactic acid fermentation is characterized by a deepest minimum entropy density acceleration and thus by a bigger variation of it as it approaches the global thermodynamic equilibrium.

The findings of this work could open the route towards other investigations focusing on the statistical thermodynamic description of glucose catabolism in human cells with special regard to entropy generation, entropy balance and entropy exchange and how these phenomena are related to the entropy density acceleration.
